# Supplementary material for: Comparison of Longitudinal and Cross-Sectional Approaches in Studies on Knowledge, Attitude and Practices Related to Non-Medical Tranquilizer Use
Source: J Clin Med. 2021 Oct 21;10(21):4827. doi: 10.3390/jcm10214827 (PMC8584824; doi:10.3390/jcm10214827)
Supplement: Supplementary file 1 [file jcm-10-04827-s001.zip › jcm-1229883-supplementary.pdf]

Comparison of Longitudinal and Cross-Sectional Approaches in Studies on Knowledge, Attitude and Practices Related to Non-Medical Tranquilizer Use

Narmeen Mallah [1,2,3], Julia Battaglia [1], Adolfo Figueiras [1,2,3] and Bahi Takkouche [1,2,*]

[1] Department of Preventive Medicine, University of Santiago de Compostela, 15782 Santiago de Compostela, Spain; narmeen.mallah@usc.es (N.M.); julia.battaglia@rai.usc.es (J.B.); adolfo.figueiras@usc.es (A.F.)

[2] Centro de Investigación Biomédica en Red de Epidemiología y Salud Pública (CIBER-ESP),
28029 Madrid, Spain

[3] Health Research Institute of Santiago de Compostela (IDIS), 15706 Santiago de Compostela, Spain

* Correspondence: bahi.takkouche@usc.es; Tel.: +34-881-812-268

Supplementary Table S1. Estimates of the association of knowledge and attitudes with any practice of non-medical tranquilizer use using cross-sectional and longitudinal approaches

Knowledge or Attitude Statement, level of agreement	Cross-sectional approach (N=847) No non-medical use (N)	Non-medical use (N)	OR (95%CI)*	Longitudinal approach (N=1343) No non-medical use (N)	Non-medical use (N)	OR (95%CI)**
Q1. I would agree to take tranquilizers in order to sleep better						
0-5	528	27	1.00	857	40	1.00
6-8	150	21	2.64 (1.43 – 4.88)	217	44	4.63 (2.17 - 9.88)
9-10	81	26	4.90 (2.66 – 9.02)	129	37	5.72 (2.43 - 13.44)
Q2. If I feel better after a few days, I will keep taking my tranquilizers even after completing the prescribed course of treatment						
0	529	35	1.00	835	62	1.00
1-5	163	23	2.22 (1.26 – 3.91)	265	31	1.66 (0.79 - 3.47)
6-10	44	16	4.79 (2.39 – 9.60)	63	28	6.33 (2.45 - 16.34)
Q3. I would take tranquilizers in order to enjoy myself with my family						
0	439	31	1.00	696	57	1.00
1-5	215	24	1.69 (0.96 – 2.99)	343	37	1.58 (0.78 - 3.19)
6-10	99	19	2.55 (1.35 – 4.83)	156	27	2.07 (0.88 - 4.82)
Q4. I would agree to take tranquilizers when I feel down and sad in order to work better						
0	380	23	1.00	597	51	1.00
1-5	254	26	1.77 (0.98 - 3,20)	417	35	1.16 (0.57 - 2.37)
6-10	116	24	3.18 (1.69 - 5.96)	177	35	2.35 (1.04 - 5.28)
Q5. Tranquilizers reduce people's control over what they do						
0-4	150	21	1.33 (0.72 - 2.44)	245	44	1.91 (0.87 - 4.2)
5-7	297	24	0.79 (0.44 – 1.42)	461	39	0.89 (0.42 - 1.89)
8-10	290	29	1.00	465	37	1.00
Q6. People taking tranquilizers are at increased risk of traffic accidents						
0-5	216	25	1.11 (0.60 – 2.03)	343	36	1.06 (0.48 - 2.33)
6-9	289	24	0.86 (0.47 – 1.56)	447	43	0.99 (0.46 - 2.1)
10	245	24	1.00	400	38	1.00
Q7. Psychotropic drugs (such as tranquilizers) may affect children's learning abilities when prescribed to them						
0-5	313	34	1.09 (0.62 – 1.90)	477	56	0.97 (0.48 - 1.97)
6-8	196	14	0.78 (0.39 – 1.56)	319	21	0.57 (0.24 - 1.38)
9-10	241	25	1.00	393	40	1.00
Q8. If I feel side effects during a course of treatment of tranquilizers, I should stop taking it as soon as possible						
0-5	346	19	0.72 (0.41 – 1.26)	542	49	0.89 (0.43 - 1.85)
6-9	165	19	1.11 (0.58 – 2.12)	256	34	1.57 (0.68 - 3.62)
10	238	26	1.00	388	38	1.00

Knowledge or Attitude Statement, level of agreement	Cross-sectional approach (N=847) No non-medical use (N)	Non-medical use (N)	OR (95%CI)*	Longitudinal approach (N=1343) No non-medical use (N)	Non-medical use (N)	OR (95%CI)**
Q9. I would take the *tranquilizers* according to the doctor's instructions						
0-7	163	14	1.00 (0.53 – 1.88)	259	22	0.88 (0.38 – 2.00)
8-9	100	12	1.42 (0.71 – 2.82)	150	28	2.59 (1.15 - 5.88)
10	480	47	1.00	768	71	1.00
Q10. If *tranquilizers* are consumed in excess, they won't work when they are really needed						
0-6	216	15	0.66 (0.35 – 1.25)	328	40	1.49 (0.73 - 3.03)
7-9	147	17	1.21 (0.66 – 2.23)	224	27	1.51 (0.66 - 3.44)
10	380	41	1.00	627	51	1.00
Q11. I prefer to keep *tranquilizers* at home in case there was a need for them later						
0-3	485	23	1.00	780	36	1.00
4-8	202	27	3.00 (1.66 – 5.43)	296	44	2.7 (1.28 - 5.70)
9-10	62	23	6.75 (3.49–13.08)	102	41	9.49 (4.05 - 22.25)
Q12. I will trust the doctor's decision if s/he decided to prescribe or not prescribe *tranquilizers*						
0-6	166	19	1.35 (0.75 – 2.46)	268	30	1.35 (0.61 - 3.01)
7-9	202	18	1.05 (0.57 – 1.93)	302	43	2.23 (1.08 - 4.6)
10	387	37	1.00	626	48	1.00
Q13. If I believe that I need a *tranquilizer* and the doctor did not prescribe it, I will get it at the pharmacy without a prescription						
0	579	47	1.00	828	84	1.00
1-5	138	14	1.31 (0.68 – 2.52)	207	13	0.65 (0.25 - 1.66)
6-10	36	12	4.20 (1.99 – 8.87)	57	23	5.5 (2.03 - 14.87)
Q14. I think that doctors often explain clearly to the patients the reasons for prescribing or not prescribing *tranquilizers*						
0-5	334	28	0.93 (0.52 – 1.67)	516	50	1.04 (0.5 - 2.16)
6-8	175	22	1.45 (0.78 – 2.71)	254	34	1.57 (0.7 - 3.54)
9-10	242	24	1.00	418	37	1.00
Q15. I think that doctors often explain clearly to the patients the instructions for the use of *tranquilizers*						
0-5	291	27	1.19 (0.66 – 2.14)	453	42	0.95 (0.46 - 1.96)
6-8	181	21	1.50 (0.80 – 2.81)	261	34	1.49 (0.68 - 3.25)
9- 10	280	24	1.00	473	45	1.00
Q16. I think that, when dispensing *tranquilizers*, the pharmacists tell the customers about the importance of correct therapeutic compliance/adherence						
0-4	182	24	0.89 (0.48 – 1.65)	298	43	1.24 (0.55 - 2.77)
5-8	388	24	0.46 (0.25 – 0.83)	578	42	0.59 (0.27 - 1.28)
9-10	178	26	1.00	298	36	1.00

:** OR adjusted for age, gender, alcohol intake and medical consultation over the phone *:** OR adjusted for age, gender, employment status and frequency of doctor consultation in case of illness.

Supplementary Table S2. Estimates of the association of knowledge and attitudes with specific practices of non-medical tranquilizer use using cross-sectional and longitudinal approaches

Knowledge or Attitude Statement	Cross-sectional approach (N= 847)						Longitudinal approach (N= 1343)			
	Shortened treatment		Stored leftover		Changed dose		Stored leftover		Changed dose	
Level of agreement	N	OR (95%CI)*	N	OR (95%CI)*	N	OR (95%CI)*	N	OR (95%CI)**	N	OR (95%CI)**
**Q1. I would agree to take tranquilizers in order to sleep better**										
0-5	7	1.00	15	1.00	14	1.00	20	1.00	7	1.00
6-8	9	3.93 (1.41, 10.95)	15	3.29 (1.55, 6.97)	7	1.52 (0.59, 3.90)	19	3.36 (1.24, 9.13)	7	4.31 (1.03, 17.93)
9-10	8	5.93 (2.04, 17.19)	17	5.84 (2.76, 12.36)	13	4.13 (1.81, 9.45)	18	4.55 (1.51, 13.74)	25	16.98 (4.67, 61.77)
**Q2. If I feel better after a few days, I will keep taking my tranquilizers even after completing the prescribed course of treatment**										
0	10	1.00	22	1.00	13	1.00	27	1.00	19	1.00
1-5	8	2.42 (0.94, 6.28)	15	2.19 (1.10, 4.36)	14	3.47 (1.59, 7.59)	20	2.66 (1.04, 6.78)	9	1.3 (0.39, 4.32)
6-10	6	5.78 (2.01, 16.69)	10	4.54 (2.02, 10.22)	7	4.33 (1.57, 11.95)	10	3.59 (0.98, 13.22)	11	4.93 (1.35, 17.94)
**Q3. I would take tranquilizers in order to enjoy myself with my family**										
0	11	1.00	19	1.00	13	1.00	30	1.00	18	1.00
1-5	6	1.97 (0.71, 5.47)	17	1.92 (0.97, 3.80)	10	1.59 (0.68, 3.70)	10	0.88 (0.31, 2.5)	11	1.1 (0.35, 3.44)
6-10	7	1.34 (0.51, 3.55)	11	2.48 (1.13, 5.44)	11	3.27 (1.37, 7.76)	17	2.63 (0.93, 7.45)	10	1.8 (0.52, 6.16)
**Q4. I would agree to take tranquilizers when I feel down and sad in order to work better**										
0	9	1.00	15	1.00	11	1.00	21	1.00	22	1.00
1-5	8	2.25 (0.85, 5.96)	14	1.37 (0.65, 2.91)	12	1.62 (0.70, 3.74)	13	1.16 (0.42, 3.21)	7	0.52 (0.16, 1.74)
6-10	6	1.02 (0.36, 2.91)	17	3.45 (1.65, 7.21)	10	2.33 (0.93, 5.82)	23	3.54 (1.27, 9.85)	10	1.22 (0.36, 4.09)
**Q5. Tranquilizers reduce people's control over what they do**										
0-4	5	0.84 (0.28, 2.52)	12	1.11 (0.52, 2.37)	11	1.85 (0.78, 4.42)	26	3.24 (1.12, 9.38)	16	2.4 (0.71, 8.05)
5-7	9	0.84 (0.33, 2.11)	16	0.81 (0.41, 1.62)	12	1.00 (0.42, 2.36)	16	0.97 (0.33, 2.89)	14	1.24 (0.37, 4.17)
8-10	10	1.00	19	1.00	11	1.00	14	1.00	9	1.00

Knowledge or Attitude Statement	Cross-sectional approach (N= 847)						Longitudinal approach (N= 1343)			
	Shortened treatment		Stored leftover		Changed dose		Stored leftover		Changed dose	
Level of agreement	N	OR (95%CI)*	N	OR (95%CI)*	N	OR (95%CI)*	N	OR (95%CI)**	N	OR (95%CI)**
Q6. People taking tranquilizers are at increased risk of traffic accidents										
0-5	6	0.77 (0.26, 2.28)	18	1.39 (0.67, 2.89)	12	0.98 (0.42, 2.29)	20	1.43 (0.5, 4.12)	9	0.98 (0.28, 3.4)
6-9	10	1.03 (0.40, 2.68)	15	0.90 (0.42, 1.91)	9	0.62 (0.26, 1.51)	20	1.13 (0.39, 3.24)	17	1.32 (0.42, 4.15)
10	8	1.00	14	1.00	12	1.00	16	1.00	13	1.00
Q7. Psychotropic drugs (such as tranquilizers) may affect children's learning abilities when prescribed to them										
0-5	4	2.38 (0.71, 7.92)	8	2.00 (0.85, 4.72)	13	0.90 (0.39, 2.04)	30	1.17 (0.47, 2.92)	18	1.14 (0.35, 3.66)
6-8	10	1.78 (0.73, 4.36)	17	1.41 (0.73, 2.73)	8	0.96 (0.38, 2.45)	4	0.26 (0.06, 1.21)	12	1.51 (0.43, 5.31)
9-10	10	1.00	22	1.00	12	1.00	20	1.00	9	1.00
Q8. If I feel side effects during a course of treatment of tranquilizers, I should stop taking it as soon as possible										
0-5	9	0.46 (0.18, 1.16)	16	0.55 (0.27, 1.12)	14	0.83 (0.37, 1.87)	18	0.53 (0.2, 1.42)	13	0.69 (0.23, 2.11)
6-9	5	0.61 (0.20, 1.80)	13	1.09 (0.51, 2.32)	9	1.09 (0.42, 2.80)	18	1.27 (0.44, 3.65)	10	1.25 (0.35, 4.4)
10	11	1.00	18	1.00	11	1.00	21	1.00	16	1.00
Q9. I would take the tranquilizers according to the doctor's instructions										
0-7	6	2.31 (0.90, 5.91)	10	1.11 (0.52, 2.35)	9	1.57 (0.69, 3.57)	17	2.03 (0.74, 5.55)	4	0.56 (0.13, 2.42)
8-9	7	2.22 (0.74, 6.60)	8	1.45 (0.64, 3.32)	6	1.34 (0.48, 3.73)	15	3.45 (1.17, 10.15)	9	2.17 (0.66, 7.14)
10	11	1.00	29	1.00	19	1.00	25	1.00	26	1.00
Q10. If tranquilizers are consumed in excess, they won't work when they are really needed										
0-6	5	0.58 (0.21, 1.65)	11	0.85 (0.41, 1.78)	10	1.18 (0.50, 2.75)	26	4.12 (1.5, 11.33)	12	1.72 (0.52, 5.66)
7-9	5	1.21 (0.42, 3.47)	11	1.22 (0.58, 2.57)	7	1.48 (0.61, 3.59)	16	3.09 (0.96, 9.93)	16	3.72 (1.16, 11.94)
10	14	1.00	25	1.00	15	1.00	12	1.00	11	1.00

Knowledge or Attitude Statement	Cross-sectional approach (N= 847)						Longitudinal approach (N= 1343)			
	Shortened treatment		Stored leftover		Changed dose		Stored leftover		Changed dose	
Level of agreement	N	OR (95%CI)*	N	OR (95%CI)*	N	OR (95%CI)*	N	OR (95%CI)**	N	OR (95%CI)**
Q11. I prefer to keep tranquilizers at home in case there was a need for them later										
0-3	7	1.00	14	1.00	11	1.00	20	1.00	8	1.00
4-8	9	2.71 (0.98, 7.48)	14	6.86 (3.11, 15.11)	10	2.05 (0.85, 4.95)	15	1.38 (0.47, 4.01)	13	3.91 (1.11, 13.83)
9-10	8	6.86 (2.37, 19.86)	19	11.78(5.54, 25.02)	12	6.08 (2.50, 14.83)	22	6.72 (2.34, 19.3)	18	13.94 (3.82, 50.85)
Q12. I will trust the doctor's decision if s/he decided to prescribe or not prescribe tranquilizers										
0-6	10	3.11 (1.20, 8.07)	11	1.25 (0.59, 2.65)	13	2.45 (1.12, 5.39)	19	2.33 (0.81, 6.69)	7	0.66(0.16, 2.75)
7-9	6	1.55 (0.53, 4.57)	14	1.46 (0.72, 2.95)	7	0.93 (0.35, 2.48)	23	2.97 (1.1, 8.03)	13	1.78(0.6, 5.24)
10	8	1.00	22	1.00	14	1.00	15	1.00	19	1.00
Q13. If I believe that I need a tranquilizer and the doctor did not prescribe it, I will get it at the pharmacy without a prescription										
0	15	1.00	27	1.00	17	1.00	38	1.00	28	1.00
1-5	4	1.05 (0.34, 3.25)	10	1.66 (0.78, 3.57)	9	2.15 (0.90, 5.14)	9	1.1 (0.34, 3.51)	4	0.66(0.15, 2.9)
6-10	5	4.40 (1.50, 12.93)	9	5.05 (2.17, 11.74)	7	6.15 (2.35, 16.05)	9	3.1 (0.81, 11.84)	7	3.2(0.76, 13.52)
Q14. I think that doctors often explain clearly to the patients the reasons for prescribing or not prescribing tranquilizers										
0-5	8	2.41 (0.85, 6.84)	17	1.06 (0.50, 2.25)	15	1.14 (0.49, 2.62)	28	4.11 (1.11, 15.24)	15	0.88(0.29, 2.64)
6-8	9	0.81 (0.30, 2.24)	17	2.07 (0.97, 4.41)	9	1.33 (0.53, 3.37)	25	7.87 (2.04, 30.36)	8	1.04(0.3, 3.67)
9-10	7	1.00	13	1.00	10	1.00	4	1.00	16	1.00
Q15. I think that doctors often explain clearly to the patients the instructions for the use of tranquilizers										
0-5	6	2.78 (0.91, 8.56)	16	1.24 (0.59, 2.60)	15	1.53 (0.66, 3.52)	23	2.26 (0.75, 6.83)	11	0.74(0.22, 2.45)
6-8	11	1.24 (0.47, 3.27)	16	2.01 (0.95, 4.26)	8	1.32 (0.51, 3.44)	25	4.65 (1.52, 14.23)	12	1.78(0.57, 5.59)
9- 10	7	1.00	14	1.00	10	1.00	9	1.00	16	1.00

Knowledge or Attitude Statement	Cross-sectional approach (N= 847)						Longitudinal approach (N= 1343)			
	Shortened treatment		Stored leftover		Changed dose		Stored leftover		Changed dose	
Level of agreement	N	OR (95%CI)*	N	OR (95%CI)*	N	OR (95%CI)*	N	OR (95%CI)**	N	OR (95%CI)**
Q16. I think that, when dispensing tranquilizers, the pharmacists tell the customers about the importance of correct therapeutic compliance/adherence										
0-4	11	2.96 (1.00, 8.74)	13	0.72 (0.34, 1.53)	12	1.95 (0.82, 4.66)	19	1.4 (0.47, 4.2)	14	0.83(0.27, 2.59)
5-8	8	1.23 (0.39, 3.84)	16	0.43 (0.21, 088)	11	0.69 (0.29, 1.63)	23	0.8 (0.28, 2.25)	7	0.33(0.1, 1.09)
9-10	5	1.00	18	1.00	11	1.00	15	1.00	18	1.00

*****: OR adjusted for age, gender, alcohol intake and medical consultation over the phone ******: OR adjusted for age, gender, employment status and frequency of doctor consultation in case of illness.
